# Co-designing psychosis simulated patient scenarios with mental health stakeholders for pharmacy curricula

**DOI:** 10.1007/s11096-023-01622-9

**Published:** 2023-07-28

**Authors:** Tina X. Ung, Claire L. O’Reilly, Rebekah J. Moles, Sarira El-Den

**Affiliations:** School of Pharmacy, The University of Sydney, Pharmacy and Bank Building A15, Science Rd, Camperdown, Sydney, NSW 2006 Australia

**Keywords:** Co-design, Content validation, Mental health education, Pharmacy, Psychosis, Simulation

## Abstract

**Background:**

Pharmacists need knowledge and confidence to support people living with mental illness. Evidence-based educational materials for pharmacy students to provide psychosis care is limited.

**Aim:**

To co-design, content validate and pilot-test, with mental health stakeholders, simulated patient scenarios to educate and assess students in providing psychosis care.

**Method:**

Mental health consumers were invited to co-design three simulated patient scenarios (first-episode psychosis, carer of someone living with schizophrenia, non-adherence to antipsychotics), guided by published and psychometrically-tested materials. A panel of mental health stakeholders participated in two rounds of content validation (RAND/UCLA appropriateness model). Round 1 involved individual survey completion to calculate item content validity index (I-CVI) for relevance/clarity, content validity ratio for essentiality and overall scale content validity index (S-CVI/Ave and S-CVI/UA) scores for each scenario. Scores analyses and feedback comments informed revisions. Round 2 involved a panel meeting to discuss revisions and finalise content. The scenarios were then pilot-tested with pharmacy students.

**Results:**

Two consumers participated in co-design, nine stakeholders in content validation. All items showed excellent content validity for relevance/clarity. Eleven items were revised for essentiality, discussed, then re-rated at the panel meeting for consensus. The scenarios were pilot-tested with pharmacy students (n = 15) and reported to be realistic and relevant to future practice, contributing to students’ confidence in supporting people experiencing mental health symptoms or crises.

**Conclusion:**

Partnering with mental health stakeholders has enabled co-design of authentic, content valid educational materials for pharmacy students to provide psychosis care, in preparation for future provision of mental health support.

**Supplementary Information:**

The online version contains supplementary material available at 10.1007/s11096-023-01622-9.

## Impact statements

- Co-design through collaboration with people with lived experience of mental illness and healthcare professionals allows for the development of authentic psychosis care assessment materials for pharmacy curricula.
- Simulated patient role-play assessments are well-received by pharmacy students, who value opportunities to practise newly-acquired Mental Health First Aid skills and build their confidence in providing psychosis care.
- Authentic educational materials, co-designed with mental health stakeholders, may be adapted for continuing professional development activities designed to upskill practising pharmacists in providing mental health support.

## Introduction

Mental illness is a leading cause of global health-related burden; there is urgency to strengthen mental healthcare [1]. Healthcare professionals require relevant knowledge and skills to support mental health consumers and their carers [2]. People living with mental illness are “experts by experience”; incorporating this unique experiential knowledge via experience-based co-design (EBCD) [3] is pivotal in developing and delivering authentic, person-centred mental health services to meet their needs, and educational needs of healthcare professionals [4, 5].

While personal experiences of mental health consumers/carers enrich healthcare education via learning activities (e.g. lectures, workshops), there is limited involvement of mental health consumers/carers in educational design, and limited direction on methodological approaches in this area [6, 7]. In nursing, a consumer-academic role partnering with faculty to develop and teach a psychopathology course from a consumer’s perspective (alongside the traditional medical approach) for postgraduate psychiatric nursing students was positively perceived by students and faculty members and upon reflection, impactful on clinical practice [8, 9]. In medicine, mental health consumers were involved in a steering committee with academics to jointly plan, develop, implement and evaluate curriculum regarding psychiatric interviewing skills, demonstrating feasibility [10]. Consumers with chronic disease (including mental illness) and their families participated in planning teams to develop workshop learning objectives and activities for occupational therapy, pharmacy, nursing, medicine, dietetics, social work and physical therapy students, based on what they thought was important for students to learn [4]. Another approach to co-designing health education with consumers incorporated arts and humanities-based teaching methodologies (e.g. storytelling, visual mind maps) [6]. Nonetheless, co-design with consumers is lacking in pharmacy education. A recent systematic review of consumer interactions with student pharmacists [11] found that while studies involved consumers in education delivery, minimal studies report on consumer involvement in content design; there is a need to further engage consumers to co-design curricula to ensure authenticity and relevance to practice. This is a significant research gap; pharmacists cite lack of education, skills, experience and confidence as barriers to caring for people living with mental illness [12–15]. Another attributable barrier to providing mental health support is stigma; perceived stigma by healthcare professionals can negatively impact patient help-seeking and access to care, consequently impeding recovery [16–18].

Effective strategies to reduce stigma include contact with mental health consumers [19]. Mental Health First Aid (MHFA) training [20] trains participants to approach and assist people experiencing mental health problems/crises [21], increasing confidence and reducing stigma. MHFA training has been widely delivered and evaluated for the general public [22] and tertiary healthcare students [20, 23]. In tertiary pharmacy programs, mental health consumers/carers have been involved in the delivery of mental health education [17, 19, 24], including assessment of MHFA skills via simulated patient (SP) role-plays [25–27]. SP role-plays are an effective and well-received method of education and assessment in pharmacy practice [28]. In pharmacy education, this pedagogy can reduce stigma, improve attitudes and increase willingness and confidence to support people living with mental illnesses (e.g. depression, anxiety) and for people experiencing mental health crises (e.g. suicide) [25, 26]. Qualitative research indicates that both consumers and students benefit from role-play participation [25].

Nonetheless, pharmacists’ knowledge and literacy of psychotic illnesses is lacking; symptoms of schizophrenia are less easily recognised by pharmacists than depression [29]. People living with schizophrenia die at least 13–15 years earlier than the general population, mostly due to associated comorbidities [30]. Pharmacists can play crucial roles in psychosis care; fulfilment of this important role requires training and confidence.

### Aim

The aim of this project was to partner with mental health stakeholders (people with lived experience of mental illness and health professionals) to co-design, content validate and pilot-test SP scenarios for pharmacy education in psychosis care.

### Ethics approval

This project was approved by The University of Sydney Human Research Ethics Committee (Project No.2020/776 and 2015/626); conducted with reference to Ethics Guidelines for Internet-mediated Research [31]. Participants provided written consent.

## Method

The project involved three phases: Co-design (Phase 1) of educational materials (SP scenarios and associated marking rubrics) by mental health consumers/carers and the research team, content validation (Phase 2) of the educational materials by a panel of mental health stakeholders, followed by mixed-methods pilot-testing for face validity (Phase 3) among final year Master of Pharmacy (MPharm) students.

This manuscript was prepared according to the Strengthening the Reporting of Observational Studies in Epidemiology (STROBE) cross-sectional reporting guidelines [32].

### Participant recruitment

Recruitment of mental health consumer/carer participants for Phases 1 and 2 was facilitated by One Door Mental Health, a not-for-profit organisation that advocates for and trains mental health consumers/carers to speak publicly about their lived experience [33]. The research team aimed to recruit up to four mental health consumers/carers for each phase. Eligibility criteria were: people aged over 18 years living with, or caring for someone living with, mental illness. Eligible mental health consumers/carers participated exclusively in either Phase 1 or 2.

Mental health professionals (psychiatrists, psychologists) and pharmacists known to the research team were invited to join the consumers/carers in Phase 2. Following content validation process recommendations [34, 35], the research team aimed to recruit up to 10 panellists for Phase 2, including a diverse representation of mental health stakeholders.

All final year MPharm students (n = 50) at The University of Sydney, who had completed a mental health unit of study, MHFA training and enrolled in a Professional Practice unit of study, were placed into five groups of 10 for Phase 3. All students were invited to participate in a focus group in the week following the simulations.

### Phase 1 co-design

Mental health consumers/carers were invited to meet with three researchers to co-design three scenarios (and associated marking rubrics), involving people and carers of people experiencing symptoms, illnesses and crises whereby psychosis is a key feature (e.g. schizophrenia). In this manuscript, these are referred to as educational materials. The scenario topics were intended to provide a variety of psychosis-related experiences that pharmacists may encounter in practice: a person experiencing first-episode psychosis, a carer of a person living with schizophrenia, a person who is non-adherent to antipsychotics and at risk of relapse. For each scenario, the co-design panel discussed demographics (gender/age), presenting signs/symptoms, medical/social history, opening lines, responses to anticipated questions, and pharmacists’ role in providing support. The researchers drafted the educational materials based on the audio-recorded discussions, guided by published, psychometrically-tested rubrics previously used in curricula [27]. Further development was conducted via an iterative process of listening to the audio-recording for finer details, followed by discussions and modifications until consensus was reached amongst researchers.

### Phase 2 content validation

Mental health stakeholders were invited to content validate the educational materials, through a two-round modified Delphi process, guided by the RAND/UCLA appropriateness model [35, 36]. This entailed an initial individual rating round followed by a meeting to discuss results to attain consensus [35, 36]. Each Phase 2 participant individually completed an online survey, rating every item (each SP profile/scenario section, action items in the associated marking rubric) of the educational materials for relevance, clarity, essentiality, with opportunity to provide written comments. Survey data were collected and managed using the REDCap electronic data capture tool hosted at The University of Sydney [37]. Relevance and clarity were rated on a four-point scale (1 = not relevant/clear, 2 = unable to assess relevance/clarity without item revision, 3 = relevant/clear but needs minor alteration, 4 = very relevant/clear and succinct), enabling quantitative calculation of item content validity index (I-CVI) scores [34, 38–43]. A multi-rater agreement score (Kappa co-efficient) was calculated for I-CVIs, yielding a degree of agreement beyond chance [38, 41, 44, 45]. Essentiality was rated on a three-point scale (1 = not essential, 2 = useful but not essential, 3 = essential), to calculate content validity ratio (CVR) [44, 46–48]. Overall scale-level average content validity index (S-CVI/Ave) and scale-level content validity index by universal agreement (S-CVI/UA) scores for each scenario were calculated [41, 44]. Analyses of I-CVI, CVR, S-CVI/Ave and S-CVI/UA scores, alongside free-text feedback comments, informed item revisions. The revised materials were disseminated to Phase 2 participants in preparation for Round 2, involving a panel meeting with all members of the research team, facilitated by TU via Zoom video conference [49] due to Covid-19 restrictions. The meeting focussed on revisions of items with low content validity. Revised items were presented to the panel and re-rated via live Zoom polls (“repolled”). The panel meeting was audio-recorded, transcribed to inform final revisions of educational materials, then disseminated again to Phase 2 participants for final feedback before pilot-testing.

### Phase 3 pilot-testing for face validity

As part of the Professional Practice unit of study, MPharm students attend two 2.5 h tutorials per week; five groups of 10 students running concurrently. While the simulations were developed for face-to-face in-class delivery, pilot-testing was conducted on Zoom due to Covid-19 restrictions. One researcher (TU, who is MHFA-trained) undertook the dual role of SP and tutor to pilot-test the scenarios with students over three sessions (1.5 weeks). In each session, one student from each group was randomly allocated to role-play with TU, who also assessed the student using the developed marking rubric while the remaining nine classmates observed. Immediately after each simulation, the role-playing student self-assessed their performance, scoring themselves either full/partial/no marks for each rubric item, followed by feedback from TU and debrief discussion with all students. This process took ~ 30 min; TU then moved to the next virtual classroom, repeating this process five times per session. One scenario was pilot-tested during each of the three sessions, with each of the five groups, totalling 15 role-plays for scoring. Audio-recorded focus groups were conducted on Zoom in the week following the final session, allowing students to voice their perspectives on the scenarios. Audio-recordings were transcribed, then listened to multiple times by one researcher (TU). Student quotes were discussed amongst the research team to discern whether the scenarios were face valid, acceptable, and relevant.

## Results

### Phase 1 co-design

Two mental health consumers attended the Phase 1 co-design panel meeting in April 2021 (166 min). Table 1 provides examples of co-design panel quotes that informed scenario medical/social histories.

**Table 1:** Examples of co-design panel quotes that informed scenario medical/social histories

Scenario	Co-design panel quotes	Resulting scenario information
1	“I found marijuana more relaxing…It was the only way to keep the voices out of my head” [C1^a^]	Sleeping troubles have worsened since leaving home–you self-medicate with marijuana (1 joint before bed) and beer. You smoke 1 pack of cigarettes daily, and drink at least 6 cups of coffee or energy drinks every day
	“Alcohol makes the medication go down nicer” [C1]	
	“Cigarette addictions are prevalent in schizophrenia people” [C2^b^]	
2	“She's doing so much caring and that, she couldn't afford to be married…She's looking after her parents. She's looking after the younger brother. If she had a family, how could she fit them in?” [C1]	You were seeing someone previously and wanted to start a family, but your relationship fizzled as you “*couldn’t find the time for him*”
3	“I went down from like in excess of 200 kilos down to like 140…It was an enormous amount of weight (lost) when I came off the Zyprexa® [olanzapine]” [C2]	Because you know that Zyprexa® (olanzapine) has caused your weight gain, you have been weaning yourself off it for the past few weeks, especially when you know you need to wake up early the next day for your wife’s antenatal classes…You are also very curious to know what it is like to be “normal”, without taking the Zyprexa® (olanzapine)
	“I used to miss meds to wake up in time…if I had early morning appointments” [C2]	
	“I’ve done it before for two weeks…I wanted to find out what it would feel like to be normal” [C1]	

^a^C1 = consumer/carer 1

^b^C2 = consumer/carer 2

After further development of educational materials, including rubric items with pass/fail assessment criteria, the educational materials contained a total of 83 items: 30 in scenario 1, 27 in scenario 2, 26 in scenario 3.

### Phase 2 content validation—round 1 survey

Nine mental health stakeholders (four mental health consumers/carers, two pharmacist researchers, one psychiatrist, one academic psychologist, one counsellor/mental health social worker) participated in Phase 2, Round 1. For a panel of nine judges, minimum I-CVI (relevance, clarity) and CVR (essentiality) for excellent content validity is 0.78 [34, 41]. Each item in all three scenarios demonstrated excellent content validity, with I-CVIs 1.00 (n = 59) or 0.89 (n = 24) for relevance and 1.00 (n = 65) or 0.89 (n = 18) for clarity. Kappa co-efficients exceeded 0.74, indicating excellent agreement beyond chance [38]. CVRs ranged 0.11–1.00; 11 items generated CVR < 0.78, indicating poor content validity for essentiality. Overall scale-level scores required a minimum S-CVI/Ave of 0.9 and S-CVI/UA 0.8 for excellent content validity [41, 44]. Overall S-CVI/Ave for each scenario were excellent (0.96 or higher), S-CVI/UA ranged 0.63–0.89 (Table 2).

**Table 2:** Average and universal agreement scores per scenario (n = 9 stakeholders)

Scenario	Relevance S-CVI/Ave^a^	Relevance S-CVI/UA^b^	Clarity S-CVI/Ave	Clarity S-CVI/UA	Average CVR^c^
1	0.96	0.63	0.96	0.67	0.89
2	0.97	0.70	0.99	0.89	0.84
3	0.98	0.81	0.98	0.81	0.88

^a^S-CVI/Ave = scale-level average content validity index; ^b^S-CVI/UA = scale-level content validity index by universal agreement; ^c^CVR = content validity ratio

### Phase 2 content validation–round 2 panel meeting

Eight of the nine Round 1 participants attended the Round 2 meeting in September 2021 (108 min). Recommended revisions for the 11 items with low CVRs were presented and repolled, with discussions until consensus was reached. Supplementary material 1 shows the 11 revised items and CVRs in the two rounds.

No further suggestions were provided when the finalised materials were emailed to Phase 2 participants. Supplementary material 2 provides finalised synopses of the SP presentations. The final marking rubric for scenario 1 contained 11 items, scenario 2 - 10 items, scenario 3 - 9 items. Each rubric item was equally weighted and scored out of 2 (2 = full marks, 1 = partial marks, 0 = no marks). As agreed upon by the Phase 2 panel, to pass each scenario a student needed to score at least 50% and fulfil all pass/fail criteria for the scenario.

### Phase 3 pilot-testing for face validity

The scenarios were pilot-tested in October 2021, with five students per scenario. Mean student score was 87% (Standard deviation (SD) = 9) for scenario 1, 86% (SD = 7) for scenario 2, 91% (SD = 5) for scenario 3. One student did not fulfil pass/fail criteria for scenario 1, all students fulfilled pass/fail criteria for scenarios 2 and 3. Two focus groups (n = 2, n = 3) were conducted (28 min, 31 min). Students highlighted that scenario 1 (first-episode psychosis) particularly stood out: *“…really interesting, because it just exposed you to something so extreme, but it's something that could happen.”* [FG1a] Students found all three scenarios realistic: *“It was really good, because with the whole acting…I could feel the pressure and I could feel the situation as if I was actually in the pharmacy trying to figure it out.”* [FG2a] Furthermore, students reflected on how the authenticity of the scenarios and assessment method positively impacted their confidence: *“It actually makes us confident enough to like talk in a situation like if we got any case…We can provide information to people…It increases our confidence level a lot.”* [FG2b].

## Discussion

### Statement of key findings

This project showcases partnership with mental health stakeholders to co-design and content validate authentic educational materials for pharmacy students, successfully pilot-tested, positively received, and relevant to future practice.

### Interpretation

Concurring with EBCD principles [3], lived experiences of mental health consumers and professionals were integral to the development of the educational materials [50, 51]. Similar to previous co-design studies [52–54], consumers met to brainstorm ideas, further developed by the research team. In contrast to previous co-design research where stakeholders reviewed one to two elements of developed materials [55], all content was reviewed by each stakeholder during content validation. As recommended by previous co-design researchers [50, 51], next steps of this research will continue involving mental health consumers, in formal delivery and evaluation of the content and face-valid educational materials in the classroom.

Content validation scores revealed that every item across all scenarios demonstrated excellent content validity for relevance and clarity. When calculating kappa coefficients (k) for I-CVI, the k value for each item was similar to its pertaining I-CVI. This is congruent with Polit et al.’s appraisal and recommendations for reporting content validity [56]; as the number of rating experts increases, the probability of chance agreement decreases, hence why I-CVI and k values converged with a panel of nine stakeholders. As suggested, “any I-CVI greater than 0.78 would fall into the range considered excellent, regardless of the number of experts” [56]. All scenario items scored 1.00 or 0.89 for relevance and clarity, affirming excellent content validity.

As recommended by Polit and Beck [41], both S-CVI/Ave and S-CVI/UA were computed. S-CVI/Ave for each scenario exceeded the minimum 0.9 for excellent content validity. Relevance S-CVI/UA for scenarios 1 and 2 and clarity S-CVI/UA for scenario 1 were below the minimum value of 0.8, however it is noted that “the S-CVI/UA calculation method is overly stringent” [41], and for a scale to be judged with excellent content validity, it contains items with I-CVIs of 0.78 or higher and S-CVI/Ave 0.90 or higher, congruent with each of the scenarios [56].

In preparation for the Round 2 panel meeting, SP demographics for all three scenarios required revision, indicated by low CVRs (0.11 or 0.33). It was agreed at the Round 2 meeting that a person (whether a class tutor/researcher as in this project, or trained actor in future studies) of any gender could role-play all SPs, and age ranges were widened. These revisions highlight the importance of SP profiles and considerations during actor recruitment, to be more inclusive and ultimately more representative of the diverse range of consumer demographics that healthcare professionals encounter. This also highlights to future healthcare professionals, especially role-playing students in the formal evaluation of this project, the importance of not stereotyping and to expect to encounter a broad range of consumers. Specific details regarding the SP’s physical attributes (clothing, makeup) were also added to further illustrate physical presentations and to aid future actor preparation and training.

During pilot testing, mean student scores were similar across the diverse psychosis-related scenarios, allowing students to demonstrate a range of MHFA skills. The highest mean score was for scenario 3 (non-adherence to antipsychotic medicine). This may correlate with pharmacists’ expertise in providing information and advice about pharmacological management of illnesses. Although this scenario scored highest, students shared in focus groups that all scenarios were engaging, interesting, and relevant to practice. These comments mirror previous research demonstrating a positive effect of SP role-plays on pharmacy students’ confidence in providing MHFA [25, 26].

### Strengths, weaknesses and further research

A significant strength of this project was partnering with a broad range of mental health stakeholders, minimising tokenistic input, allowing for representation to reflect lived experiences from both personal and clinical perspectives in developing authentic educational materials to simulate real-life practice. Phase 1 was limited to the involvement of two consumers, however their lived experiences significantly informed the design of each scenario (Table 1). A further four consumers/carers were involved in Phase 2 to include a variety of views. Phases 2 and 3 were conducted during the Covid-19 pandemic, with the Round 2 panel meeting and pilot-testing occurring online. An important EBCD consideration is dynamics of panel member interactions, which may have been influenced by the online environment. University online learning during the pandemic may have hindered fidelity of the scenarios and student engagement and experience during pilot-testing, which may have also been impacted by the researcher undertaking the dual role of SP and class tutor providing performance feedback. It is recommended that future research is conducted in-person, with trained actors role-playing SPs, alongside a mental health consumer educator and tutor providing performance feedback. It is also recommended that test re-test and interrater reliability analyses are conducted on the developed marking rubrics [27]. While no specific criteria were used to establish face validity, acceptability and relevance of the educational materials, these perspectives were elicited during rich focus group discussions. As per systematic reviews exploring consumer co-design in healthcare curricula [57, 58], it is furthermore recommended to explore the impact of MHFA training, SP scenarios and consumer participation in their design, delivery and evaluation on knowledge, skills and confidence in providing mental healthcare, via a pre-post study [53].

## Conclusion

This project provides a detailed account of a methodological approach, providing future direction and guidance on engaging people with lived experience of mental illness and mental health stakeholders in co-design of educational materials for healthcare curricula. Through co-design with mental health consumers and content validation with mental health stakeholders, three authentic SP scenarios were developed to educate and assess pharmacy students in providing psychosis care. This educational material allows future MHFA-trained healthcare professionals to practise their skills in confidently and effectively providing psychosis care and supporting people living with psychotic illnesses.

### Supplementary Information

Below is the link to the electronic supplementary material.

Supplementary file1 (DOCX 16 kb)
