# Supplementary material for: Co-designing psychosis simulated patient scenarios with mental health stakeholders for pharmacy curricula
Source: Int J Clin Pharm. 2023 Jul 28;45(5):1184–91. doi: 10.1007/s11096-023-01622-9 (PMC10600032; doi:10.1007/s11096-023-01622-9)
Supplement: Supplementary file 1 — Supplementary file1 (DOCX 16 kb) [file 11096_2023_1622_MOESM1_ESM.docx]

**Supplementary material 1.** Revised items and content validity ratios (CVRs).

| **Original item and CVR**  **(Round 1)** | **Revised item and CVR**  **(Significant revisions bolded)**  **(Round 2)** |
| --- | --- |
| Scenario 1 | |
| Background:  Your pharmacy assistant alerts you to a customer who has been standing and staring at the ear care products for the past 30 minutes.  CVR=0.11 | **You notice** a customer in the pharmacy who has been standing and staring at the ear care products **for a while.**  CVR=1.00 |
| Gender/Age: Male or Female/19-20 years  CVR=0.11 | **Gender/Age: Any/19-25 years**  **Physical attributes: Messy hair and clothes**  CVR=1.00 |
| Opening line:  “Why are there so many ear plugs here?! I just need some that work!”  CVR=0.33 | [responding to auditory hallucinations]  “Stop calling me an idiot, I’m trying to get some help with all of these ear plugs!”  CVR=0.00 |
|  | (Further discussion led to retainment of original; repolled)  CVR=0.75* |
| Scenario 2 | |
| Background:  A customer approaches the dispensary counter, whom you recognise as someone who purchased a sleeping tablet, Restavit® (doxylamine), from you last week.  CVR=0.11 | **A very tired-looking** customer approaches the dispensary counter and **hands you a repeat prescription**. You recognise this person as someone who purchased a sleeping tablet, Restavit® (doxylamine), from you last week.  CVR=1.00 |
| Gender/Age: Female/30-40 years  CVR=0.11 | **Gender/**Age: **Any/**30-40 years  CVR=0.50 (Further discussion led to widening of age range; repolled) |
|  | Gender/**Age:** Any/**30’s-50’s**  **Physical attributes: No makeup, wearing scrubs, looks tired**  CVR=1.00 |
| Signs/symptoms:  Overwhelmed, tired, exhausted, worn out, stressed.  CVR=0.56 | Overwhelmed, tired, exhausted, worn out, stressed. **Yawning, trying to keep eyes open to stay awake.**  CVR=1.00 |
| Response to anticipated question:  *Tony is doing great, his schizophrenia is well-managed but he hasn’t been able to find work.*  CVR=0.56 | *Tony is doing great, his schizophrenia is well-managed but he hasn’t been able to find work* ***and his meds make him so sleepy.***  CVR=1.00 |
| Response to anticipated question:  *After this I need to do my weekly grocery shop, drive my parents and brother to their appointments, then go back home to do the housework and prepare dinner before my night shift tonight.*  CVR=0.56 | *After this I need to do my weekly grocery shop, drive my parents and brother to their appointments, then go back home to do the housework and prepare dinner* ***for my brother*** *before* ***I head out for*** *my night shift tonight.*  CVR=1.00 |
| Response to anticipated question:  *I used to go out on Friday nights for dinner and drinks with workmates, and Sunday morning jogs but I’ve had to give them up so I can care for my brother or sleep off a night shift.*  CVR=0.56 | (Further discussion led to retainment of original; repolled)  CVR=1.00 |
| Scenario 3 | |
| Gender/Age: Male/40’s  CVR=0.33 | **Gender/**Age: **Any/**40’s  **Physical attributes: Overweight, wearing trackpants**  CVR=0.75 |
| Signs/symptoms:  Appears distracted, avoiding eye contact  CVR=0.56 | You appear distracted, **gazing off to the side** and avoiding eye contact with the pharmacist.  CVR=0.50 (Further discussion led to more detail about SP’s appearance; repolled) |
|  | You appear distracted, gazing off to the side and avoiding eye contact with the pharmacist. **Because of your weight gain, you feel most comfortable wearing tracksuits**.  CVR=0.75 |

*****Minimum CVR for panel of eight judges=0.75 [34, 41].

**Supplementary material 2.** Finalised synopses of simulated patient (SP) presentations.

**Scenario 1: First episode psychosis**

A 19-25 year-old customer, wearing messy clothes with messy hair, is frustrated by the range of ear care products in the pharmacy. The customer’s facial expressions change often, clenching their fists and shuffling their feet. The customer is mumbling and talking to themselves.

Opening line: “Why are there so many ear plugs here?! I just need some that work!”

**Scenario 2: A carer of someone living with schizophrenia**

A very tired-looking customer in their 30’s-50’s, wearing scrubs and no makeup, approaches the dispensary counter with a repeat prescription. This person purchased a sleeping tablet from the pharmacy last week. The customer appears exhausted and stressed, yawning and struggling to keep their eyes open to stay awake.

Opening line: “Could I please have this repeat for Seroquel® (quetiapine) filled for my brother, and a packet of No-Doz® (caffeine + nicotinic acid)?”

**Scenario 3: A person who is non-adherent to antipsychotics and at risk of relapse**

A frequent customer in their 40’s collects weekly Webster packs from the pharmacy. The customer is overweight, wearing trackpants, and appears distracted, avoiding eye contact with the pharmacist.

Opening line: “Hello, I’m here to collect my next Webster pack please.” (SP gives pharmacist the previous week’s Webster pack, where the Wednesday, Friday and Saturday night tablet doses of their antipsychotic medicine are untouched.)
